# Serious child and adolescent behaviour disorders; a valuation study by professionals, youth and parents

**DOI:** 10.1186/s12888-017-1363-6

**Published:** 2017-06-02

**Authors:** Karin M. Vermeulen, Daniëlle E. M. C. Jansen, Erik Buskens, Erik J. Knorth, Sijmen A. Reijneveld

**Affiliations:** 1 Department of Epidemiology, unit HTA, University of Groningen, University Medical Center Groningen, P.O. Box 30.001, 9700 RB Groningen, The Netherlands; 2 Department of Health Sciences, University of Groningen, University Medical Center Groningen, Groningen, The Netherlands; 3 Department of Special Needs Education and Youth Care, University of Groningen, University Medical Center Groningen, Groningen, The Netherlands

**Keywords:** Children, Adolescents, Valuations, Utility, Behaviour disorders

## Abstract

**Background:**

In child and youth care, quantitative estimates of the impact of serious behaviour problems have not yet been made. Such input is needed to support decision making on investments in treatment.

The aim of this paper was to elicit valuations of social and conduct disorders in children and adolescents from three different perspectives: professionals, youth, and parents.

**Methods:**

We obtained valuations from 25 youth care professionals, 50 children (age 9–10) without serious behaviour problems and 36 adolescents (age 16–17) with and without serious behaviour disorders, and 46 parents with children in the aforementioned age categories. Valuations were estimated from 18 descriptions of behaviour disorders in youth aged 9 and 15 years. Descriptions included Oppositional Defiant Disorder (ODD), Conduct Disorder (CD), and Disruptive Behaviour Disorder (DBD). Comorbid conditions were Attention Deficit Hyperactivity Disorder and substance abuse. Valuations were obtained with the EuroQol questionnaire (EQ-5D-3 L) and a visual analogue scale (VAS).

**Results:**

Valuations were generally severe; problems were by and large reported to worsen quality of life by 50% compared to being fully healthy. Professionals regarded DBD with substance abuse as most severe (VAS values 0.41 for children, and 0.43 for adolescents, i.e. less than half of normal). They rated ODD as least severe (VAS values 0.58 for children, 0.59 for adolescents). Children, adolescents and parents gave lower valuations than professionals, and had a wider range of scores, particularly at the lower end of the scale.

**Conclusions:**

Behaviour disorders pose a formidable burden from the perspectives of professionals as well as children, adolescents and parents. These results may support medical decision making to set priorities with regard to prevention and treatment based on perceived severity.

## Background

Decisions on investment in prevention and care should ideally aim at achieving the greatest possible health gains within the available means [1], but there are currently insufficient data to support such decision making in psychosocial care for children and adolescents. Valuations of perceived health-related quality of life over time are a global measure to express the benefits of care and prevention. Valuations (or ‘utility’) refer to the preferences of individuals or society for a particular set of health outcomes (e.g., a given health state or a profile of health states over time) [2]. They enable comparisons between treatment alternatives and even across disease areas. Valuations are available for a broad range of somatic health states but also for mental health states. Valuation of mental health states involves considerable challenges in measurement and interpretation [3]. Two instruments, the EQ-5D and the accompanying Visual Analogue Scale (VAS) [4], are commonly used to measure and elicit utilities based on valuations by informants like parents and professionals.

So far, no utilities have been elicited for social and conduct disorders in children and adolescents; this is unfortunate given the high prevalence and societal impact of these disorders. Data of a large population based Dutch Tracking Adolescents’ Individual Lives Survey (TRAILS) [5] indicate that 16% of adolescents are suffering from behaviour disorders, with almost equal rates for oppositional defiant (8.9%) and conduct disorder (8.6%); about 4.2% had attention deficit disorder. Prevalence rates for substance dependence (7.1%) were substantially lower than for substance abuse (29.9%). Disruptive and aggressive behaviour in early childhood may lead in the future to unfavourable developmental trajectories, dropping out of school, criminal activities, psychological disorders, and poor job performance [6–9]. These effects on health and functioning also have high financial consequences. Psychological conditions in childhood, including depression and substance abuse among American children, were estimated by Smith and Smith [8] to lead to long-term reductions in family income of about $10,400, amounting to about 20% less income than in families with non-affected children. As this loss would generally be sustained throughout the subject’s adult years, it amounts to a lifetime loss in family income of about $300,000.

Many new interventions are emerging or being applied on a much larger scale to prevent or reduce behaviour disorders. It is, however, difficult to determine whether these interventions are preferable to care as usual, i.e., whether they provide value for the additional costs involved. To assess the added value of these interventions from a broad societal perspective we need generic measures of their outcomes.

To summarise, valuation of behaviour disorders, i.e. social and conduct disorders, in children and adolescents can support decisions on investment in prevention and care. Such investments are to be considered given the high prevalence and large societal impact of these disorders. These valuations should take into account the perspectives of the various stakeholders involved. The aim of the present study was therefore to obtain valuations from different perspectives on social and conduct disorders in children and adolescents, including a VAS. We include the perspectives of professionals, children and adolescents, and parents.

## Methods

Three types of stakeholders were included in the present study: professionals working with children (4–12 years) and adolescents (12–18 years), children and adolescents themselves, and parents of children between 4 and 18 years of age. Professionals, children, adolescents and parents individually judged the same 18 descriptions of behaviour typical of children and adolescents with serious behaviour disorders.

### Construction of the vignettes

We used *vignettes* (descriptions of typical behaviours) to elicit valuations. These vignettes were constructed based on DSM-IV [10] criteria for three diagnoses: Oppositional Defiant Disorder (ODD), Conduct Disorder (CD), and Disruptive Behaviour Disorder (DBD). These three were chosen because they constitute the major diagnostic groups in children and adolescents displaying serious behaviour disorders [5, 11–14]. Concise descriptions were made for each diagnostic group. These vignettes presented the basic behaviours associated with the diagnosis in various social contexts, such as public space, home, school, and peer group, since different situations might lead to different behaviours [15, 16]. The vignettes also indicated the approximate duration of a problem.

To each of these basic descriptions, variations were added for age [17], gender, severity of problems, and comorbidity. Regarding age, similar sets of vignettes were constructed for children (age 9) and for adolescents (age 15), in which behaviours were adapted to those typical of the age categories. Regarding severity, all three diagnoses were subdivided into two levels: relatively mild and severe. Finally, to the basic descriptions were added the two most common comorbidities: [14] Attention Deficit Hyperactivity Disorder (ADHD) and substance abuse (alcohol use in the younger category and substance abuse in the older category) [5, 18, 19]. This resulted in two sets of 18 vignettes, one per age category. All vignettes started with a title mentioning the diagnosis and, where applicable, the comorbidity. The vignettes are available from the authors on request.

The face-validity and age-appropriateness of the vignettes were assessed in a pilot study by two independent experts per age group. These experts were not part of the research team, nor did they participate in the expert panels. Table 1 presents an example of a vignette.

**Table 1:** Example of vignette

Conduct Disorder/CD	
A repetitive and persistent pattern of behaviour in which the basic rights of others or appropriate societal norms or rules are violated	
Value the description of the following adolescent: A boy, 15 years of age, has shown various types of behaviour typical for CD in the past half year. In addition, he uses drugs on a regular basis. In the past 6 months, he was arrested two times for bag snatching. He often lies to obtain goods or favours or to avoid obligations. He has run away from home several times, and stayed away for a few nights. Four months ago he broke into a garden centre. He is stoned or drunk a few times a week. This behaviour is hampering his school performance. He is often absent for days and at home he is unmanageable.	

### Participants

The first group of participants was made up of professionals working with children and adolescents. A panel was recruited for both age categories, covering psychosocial care in its broadest sense, from paediatrics to police, and from special education to child and adolescent psychiatry. Eligibility criteria for the panel participants were 1) working or dealing with children and/or adolescents with serious behaviour disorders, and 2) willingness to judge a wide range of health states and behaviour aspects of children and adolescents. In addition both panels included members working in a range from junior to senior positions, offering various levels of expertise. Panel membership was exclusive; none of the professionals attended both children’s and adolescents’ meetings.

The second group included children (sixth grade; aged 9–10 years) without serious behaviour disorders, and adolescents (fourth grade; aged 16–17 years) with and without serious behaviour disorders. Children and adolescents without serious behaviour disorders were recruited via a normal primary school and a normal secondary school, respectively. Adolescents with serious behaviour disorders were recruited via a school for vocational training of adolescents with mild intellectual disability (ages 16–17 years; IQ range 60–80).

The third group, parents, were recruited through primary and secondary schools, and invited to take part in the valuation study if they had children that fulfilled one of the criteria mentioned above. All three groups of participants were independent, i.e. parents and children were not matched.

### Rating the vignettes

Respondents were asked to rate each vignette using the EQ-5D and a Visual Analogue Scale (VAS). The EQ-5D is a five-item health-related quality of life questionnaire with the following dimensions: mobility, self-care, usual activities, pain/discomfort, and anxiety/depression. Answering categories are on a three-point scale [20–24]. Values on the five dimensions were transformed into one single score, theoretically ranging from −0.59 to 1 (with lower scores representing more severe problems), using the appropriate algorithm [25].

The VAS consisted of a 10-cm line on which the anchors stood for the best and worst possible (health) states. Valuations gathered with the VAS (original values 0–100) were linearly transformed into scores from 0 to 1. Not only is this method efficient and easy to use, but it also provides meaningful valuations of relative preferences of health and treatment [25, 26].

Procedures to collect data based on the vignettes were adapted to fit each target group: professionals, children and adolescents, and parents. Valuations were generated according to a fixed protocol, and the procedure replicated that of previous valuation studies [27–29]. Following an introduction and training session, each target group performed the actual judgment task. Professionals received a booklet containing all 18 vignettes, the EQ-5D questionnaires, and the VAS forms. Vignettes were displayed in random order. Subjects could rate all descriptions of behaviours at their own pace and could look back at their ratings of previous vignettes. Data were collected from children and adolescents during a two-hour meeting in their classrooms in the presence of a researcher and a teacher. As during the session with professionals, the researcher started with an introduction and training session. After this, the actual judgment task began. Vignettes were presented to children and adolescents in random order as photos in a PowerPoint presentation. The young subjects then received the EQ-5D questionnaires and the VAS forms on paper. They could rate all descriptions of behaviour at their own pace and could look back at their ratings of previous vignettes.

Data from parents were collected individually at their homes, as too few were willing to go for this purpose to a classroom or other central setting. The further procedure with the parents was the same as with children and adolescents: parents were shown vignettes in random order as photos in a PowerPoint presentation, and could then value each behaviour description at their own pace. Data were collected without identifying characteristics of the respondents.

### Analysis and reporting

First we presented background characteristics of the various types of informants (professionals, children and adolescents, parents). Next, in the two panels of professionals (panel specialized in children, versus panel specialized in adolescents), we assessed the internal reliability computing Cronbach’s alphas and intraclass correlation coefficients (ICCs). Based on the number of tasks completed and the opinion of the professionals regarding the understandability, variability and discriminative power of the vignettes, the task was considered feasible. Overall, the professionals were positive about the aim, relevance, and procedure of the study. Cronbach’s alpha for intra-panel consistency on VAS scores was 0.88 for both panels, indicating good internal consistency. The ICCs were 0.88 (95% CI: 0.77–0.96) for the children’s panel and 0.88 (95% CI: 0.76–0.95) for the adolescents’ panel. This indicates good agreement among members in both panels.

Finally, we assessed the valuations for each vignette per category of informants (professionals, children/adolescents, parents), by computing routine descriptives (mean, standard deviation, median, and range of values). In the panel of professionals this was done for both the EQ-5D and the VAS scores; among the children/adolescents and parents this was based only on the VAS.

## Results

### Participants

Table 2 shows the background characteristics of the participants. Of the professionals, all 15 who were invited to assess children’s vignettes attended the one-day meeting. In the panel valuing adolescents’ vignettes, 10 of the 14 invited professionals participated. The group of 6th graders consisted of 50 children, the secondary school group consisted of 15 adolescents, and the adolescent group from the school for vocational training consisted of 21 people. Their demographic details can be found in Table 2.

**Table 2:** Demographic characteristics and health state of informants

Informants	Demographics		Health state	
	Age Mean (sd)	Gender-Male n (%)	VAS Mean (sd)	EQ-5D score ^b^ Mean (sd)
Professionals for 4–12 year olds (=15)	nr ^a^	3 (20)	0.88 (0.11)	0.88 (0.11)
Professionals for 12–18 year olds (*n* = 10)	nr	4 (40)	0.90 (0.09)	0.90 (0.11)
Children in regular primary education (*n* = 50)	10 (0.57)	36 (72)	0.87 (0.14)	0.86 (0.06)
Adolescents in regular secondary education (*n* = 15)	15 (1.06)	5 (33)	0.78 (0.09)	0.91 (0.04)
Adolescents in vocational training = 21)	17 (0.74)	13 (62)	0.85 (0.16)	0.84 (0.08)
Parents of children in regular primary education (*n* = 16)	43 (4.48)	5 (31)	0.85 (0.10)	0.88 (0.87)
Parents of children in regular secondary education (*n* = 15)	48 (4.71)	6 (40)	0.81 (0.11)	0.85 (0.07)
Parents of children in vocational training |(*n* = 15)	49 (8.71)	2 (13)	0.86 (0.01)	0.90 (0.62)

^a^nr = not registered

^b^ *Note.* Reference value for the Dutch general population: 0.88 [30]; higher scores represent a better health

Table 2 also shows the self-rated health status of the participants. All EQ-5D scores were within the range of the general Dutch population (0.88) [30]. VAS scores were comparable to EQ-5D scores, except for the group of adolescents following regular secondary education. This group reported lower VAS scores (0.78) compared to the other groups, and also compared to their EQ-5D scores.

The group of parents of 6th graders consisted of 16 people, the secondary school group consisted of 15 parents, and the group of parents of adolescents at the school for vocational training consisted of 15 people. Table 2 shows their demographic characteristics. All EQ-5D scores of these parents were within the range of the general Dutch population (0.88) [30]; the VAS scores were comparable to the EQ-5D scores.

### Valuations of the vignettes

#### Professionals

Table 3 presents the valuations by professionals of the vignettes describing child and adolescent behaviour. Professionals regarded ODD in children as the least severe and DBD with substance abuse as comorbidity as the most severe condition in children. With regard to the vignettes of adolescents, the professionals rated ODD as least severe, both when judged as a single behaviour state and when combined with comorbid conditions. Based on the EQ-5D, professionals rated CD with ADHD as most severe, followed by DBD with substance abuse. Based on the VAS, DBD with substance abuse was rated as most severe, followed by CD with substance abuse.

**Table 3:** Professionals’ valuations of health states in case of behaviour disorders of children and adolescents

Behaviour disorder	Children Mean (sd)	Adolescents Mean (sd)
ODD	0.58 (0.08)	0.59 (0.12)
CD	0.47 (0.14)	0.58 (0.15)
DBD	0.58 (0.08)	0.59 (0.12)
ODD+ ADHD	0.54 (0.09)	0.53 (0.12)
CD + ADHD	0.45 (0.11)	0.52 (0.17)
DBD + ADHD	0.44 (0.12)	0.44 (0.10)
ODD + substance abuse	0.50 (0.09)	0.53 (0.15)
CD + substance abuse	0.42 (0.09)	0.51 (0.18)
DBD + substance abuse	0.41 (0.10)	0.43 (0.08)

*ODD* Oppositional Defiant Disorder, *CD* Conduct Disorder, *DBD* Disruptive Behaviour Disorder, *ADHD* Attention Deficit Hyperactivity Disorder

Possible scores range from 0 (worst possible state) to1 (best possible state)

#### Children and adolescents

In general, adolescents following regular secondary education gave somewhat higher values, i.e. more favourable, compared to primary school children and adolescents following vocational training (Table 4), but without statistical significance. Both children and adolescents in regular education rated DBD with substance abuse as the worst state, followed by DBD single state, and DBD with ADHD (primary education group) or vice versa (secondary education group). Adolescents following secondary education rated DBD single state as the most severe, followed by DBD with substance abuse, and CD with substance abuse. Adolescents following regular secondary education and those following vocational training, rated as best state ODD with ADHD as comorbidity. Children following primary education, rated ODD single state as the least severe.

**Table 4:** Children and adolescents valuations of health states in case of behaviour disorders

Behaviour disorder	Regular primary education (children) Mean (sd)	Regular secondary education (adolescents) Mean (sd)	Vocational training (adolescents) Mean (sd)
ODD	0.31 (0.15)	0.52 (0.12)	0.37 (0.14)
CD	0.11 (0.09)	0.31 (0.13)	0.19 (0.15)
DBD	0.06 (0.07)	0.20 (0.13)	0.12 (0.15)
ODD+ ADHD	0.28 (0.17)	0.56 (0.09)	0.38 (0.15)
CD + ADHD	0.15 (0.11)	0.34 (0.14)	0.21 (0.15)
DBD + ADHD	0.07 (0.08)	0.18 (0.13)	0.15 (0.18)
ODD + substance abuse	0.14 (0.13)	0.41 (0.16)	0.20 (0.15)
CD + substance abuse	0.11 (0.16)	0.24 (0.13)	0.15 (0.16)
DBD + substance abuse	0.06 (0.08)	0.14 (0.13)	0.14 (0.18)

*ODD* Oppositional Defiant Disorder, *CD* Conduct Disorder, *DBD* Disruptive Behaviour Disorder, *ADHD* Attention Deficit Hyperactivity Disorder

Possible scores range from 0 (worst possible state) to1 (best possible state)

#### Parents

Table 5 shows the valuations of the parents. DBD with substance abuse was considered the worst state by all groups of parents. In the group of parents of children following regular primary education, DBD with ADHD as comorbidity had the same low value, followed by DBD single state. In parents whose children followed regular secondary education, as well as in those whose adolescents followed vocational training, DBD single state was considered the second worst state, followed by either CD with substance abuse (regular secondary education) or DBD with ADHD as comorbidity (vocational training). According to parents with children following regular primary education, as well as those with children following vocational training, ODD with ADHD as a comorbidity was considered the least severe state. However, for parents with adolescents following regular secondary education ODD single state was considered least severe.

**Table 5:** Parents’ valuations of health states in case of behaviour disorders

Behaviour disorder	Regular primary education (children’s parents) Mean (sd)	Regular secondary education (adolescents’ parents) Mean (sd)	Vocational training (adolescents’ parents) Mean (sd)
ODD	0.52 (0.080)	0.49 (0.12)	0.485 (0.14)
CD	0.36 (0.125)	0.23 (0.12)	0.269 (0.13)
DBD	0.28 (0.122)	0.12 (0.12)	0.190 (0.14)
ODD+ ADHD	0.53 (0.096)	0.44 (0.16)	0.519 (0.14)
CD + ADHD	0.36 (0.121)	0.24 (0.15)	0.315 (0.12)
DBD + ADHD	0.22 (0.131)	0.13 (0.12)	0.198 (0.14)
ODD + substance abuse	0.38 (0.155)	0.35 (0.15)	0.373 (0.15)
CD + substance abuse	0.30 (0.147)	0.21 (0.13)	0.240 (0.13)
DBD + substance abuse	0.22 (0.131)	0.11 (0.11)	0.165 (0.13)

*ODD* Oppositional Defiant Disorder, *CD* Conduct Disorder, *DBD* Disruptive Behaviour Disorder, *ADHD* Attention Deficit Hyperactivity Disorder

Possible scores range from 0 (worst possible state) to1 (best possible state)

### Effects of age, comorbidity, and gender

Valuations across the different descriptions were mostly similar for children and adolescents, especially with regard to the descriptions of DBD and ODD. With regard to CD, the ratings for adolescents were slightly higher (representing less severe problems). Valuations were also similar for boys and girls. Substance abuse had more impact on the valuations than did ADHD. This was consistent across diagnoses. The highest VAS scores were given for the descriptions without comorbidity, followed by those with ADHD as comorbidity, and finally the descriptions that included substance abuse as a comorbidity. None of the differences found were significant.

## Discussion

The aim of the present study was to obtain valuations from different perspectives on social and conduct disorders in children and adolescents. The perspectives were those of professionals, children and adolescents, and parents. We found low valuations for all conduct disorders and comorbid conditions, indicating a high burden of disease which decreased quality of life by an average of about 50%. We cannot directly compare our valuations with other studies on adolescents, as ours is the first study to elicit valuations with regard to serious behaviour disorders of children and adolescents. However, comparisons with valuations of somatic conditions in the adult population are possible. Our valuations as obtained for ODD and DBD in adolescents, as well as those for ODD, DBD, and CD in children, are comparable to those obtained for multiple sclerosis, fractured skull, and intracranial injuries in adults [31]. CD in adolescents was valued in a way similar to severe chronic obstructive pulmonary disease (COPD) in adults [31].

Compared to known values in mental health, our values were in the same range as those for moderate and severe depressive episodes in adults. Sapin et al. [32] used the EQ-5D in a population of patients with major depressive disorders and showed a mean pre-treatment score of 0.33. Bipolar affective disorder, when untreated, had a utility weight of 0.60, schizophrenia of 0.47. Obsessive-compulsive disorder, however, resulted in a utility of 0.87. All these comparisons indicate that overall, severe behaviour disorders in children and adolescents represent a serious problem, which the current study confirms by formal estimates. Yet some previous studies, using other measures of effect, also revealed a rather high burden of disease for these disorders. Strijker et al. [18] used the Child Behaviour Checklist and found a ranking of behaviour disorders which largely corroborates our findings, although their study was based on different groups. Their analyses, based on multi-dimensional scaling techniques, show that social problems were ranked as more favourable (i.e. less severe) than problems of a more aggressive and delinquent nature. Although different measurements and sampling approaches were involved, this study confirms our grading of the impact of various problems.

With regard to differences between types of raters (children, adolescents, parents and professionals) we found that, overall, valuations of professionals were a bit more favourable than those of children, adolescents and parents. Within the group of youth, children attending regular primary education tended to give lower valuations compared to the other two groups of youth, especially those attending regular secondary education. The difference between the professionals and the youth and their parents might partly be explained by the experience of the professionals: professionals tend to have experience with a wide range of problem-severity, whereas youth and their parents probably have more limited experience. Because professionals may be more capable of relating the problems presented to the most severe cases they have experienced they may make milder valuations. Professionals, also based on their experience, may adopt a long term perspective in which they anticipate certain changes (improvements). Moreover, others have reported that health state valuations performed by children themselves raise similar problems as in health or Quality of Life measurement, because children have limited cognitive and linguistic abilities. In addition, children may have a different attitude towards risk, or may have difficulty comprehending the concept of time or the possibility of death [33]. With regard to comorbidities, our study found that adding comorbid substance abuse to the descriptions of behaviour had a more negative impact on the valuations than adding ADHD. This was consistent across main diagnoses and across stakeholders. The study by Strijker et al. [18] showed similar aggravating effects of attention problems and substance use.

The odds of a secondary disorder, such as substance abuse, were four to six times higher in the presence of ODD. We can conclude that the order of severity seems to be consistent for the various diagnostic groups alone, and for those with ADHD and substance abuse as comorbidities.

Our study is the first to obtain valuations of severe behaviour disorders in children and adolescents. Its major strengths are its use of various informants: professionals, youth and parents, and its coverage of a broad range of disciplines and of youth ages.

Limitations of the present study include the fact that the variety in vignettes was relatively small given the range of diagnoses in this field. This relatively small number of vignettes was intentional in the current study design, i.e. the feasibility of this method within the chosen groups of participants was our primary aim. One of the implications of this choice was that we could not include different severity levels for the comorbid conditions; future studies should focus on that gap. Furthermore, it would be of great interest if useful comparisons could be made between different populations. At this point there are too many differences to enable a solid comparison. However, showing that valuing health states in this way is feasible, is an important step towards more systematic comparisons. An additional limitation regards the relatively small number of participants. To obtain normative values, larger samples will be required. In addition, it would be interesting to study the association between mental health education and valuations of mental health states. Our current data however do not provide the appropriate information to study this.

While our findings need confirmation, the valuations clearly indicate that the behaviour disorders we studied are perceived as severe. The valuations also indicate the aggravation effects of comorbidities. These findings show the need of offering care to this group, while taking into account the more general status of the child or adolescent. This may be helpful in the development of future interventions for this population.

## Conclusions

In conclusion, a standardized valuation of DSM-IV based vignettes of children and adolescents with social and conduct disorders is feasible and reliable for different populations. Moreover, the resulting valuations clearly discriminate between diagnoses and comorbidities. The findings also indicate the severe burden of these disorders. Future decisions regarding prevention and treatment for children and youth with behaviour and emotional problems should incorporate utility-based outcome assessments, leading to an efficient and equitable distribution of scarce health care resources.

## Abbreviations

ADHD: Attention Deficit Hyperactivity Disorder
CD: Conduct Disorder
DBD: Disruptive Behaviour Disorder
FFT: Functional Family Therapy
ICCs: Intraclass correlation coefficients
MST: Multisystemic Therapy
ODD: Oppositional Defiant Disorder
PMTO: Parent Management Training Oregon
TRAILS: Tracking Adolescents’ Individual Lives Survey
VAS: Visual analogue scale
